# Judicious Use of Lipid Lowering Agents in the Management of NAFLD

**DOI:** 10.3390/diseases6040087

**Published:** 2018-09-24

**Authors:** Umair Iqbal, Brandon J. Perumpail, Nimy John, Sandy Sallam, Neha D. Shah, Waiyee Kwong, George Cholankeril, Donghee Kim, Aijaz Ahmed

**Affiliations:** 1 Department of Medicine, Geisinger Medical Center, Danville, PA 17821, USA; umairiqbal_dmc@hotmail.com; 2 Drexel University College of Medicine, Philadelphia, PA 19104, USA; brandonperumpail@gmail.com; 3 Division of Gastroenterology and Hepatology, Stanford University School of Medicine, Stanford, CA 94304, USA; nionnj@gmail.com (N.J.); SSallam@stanfordhealthcare.org (S.S.); NeShah@stanfordhealthcare.org (N.D.S.); WKwong@stanfordhealthcare.org (W.K.); georgetc@stanford.edu (G.C.); dhkimmd@stanford.edu (D.K.)

**Keywords:** NAFLD, NASH, hepatic fibrosis, hyperlipidemia, statins

## Abstract

Non-alcoholic fatty liver disease (NAFLD) is the most common cause of chronic liver disease in the Western world. NAFLD encompasses a spectrum of histological features, including steatosis, steatohepatitis with balloon degeneration, and hepatic fibrosis leading to cirrhosis. In patients with advanced liver damage, NAFLD is associated with an increased risk of hepatocellular carcinoma. Diabetes mellitus, hypertension, and dyslipidemia are components of metabolic syndrome and are commonly associated with NAFLD. Cardiovascular disease is the leading cause of mortality in patients with NAFLD. Therefore, it is important to pre-emptively identify and proactively treat conditions like hyperlipidemia in an effort to favorably modify the risk factors associated with cardiovascular events in patients with NAFLD. The management of hyperlipidemia has been shown to reduce cardiovascular mortality and improve histological damage/biochemical abnormalities associated with non-alcoholic steatohepatitis (NASH), a subset of NAFLD with advance liver damage. There are no formal guidelines available regarding the use of anti-hyperlipidemic drugs, as prospective data are lacking. The focus of this article is to discuss the utility of lipid-lowering drugs in patients with NAFLD.

## 1. Introduction

**Figure 1:**
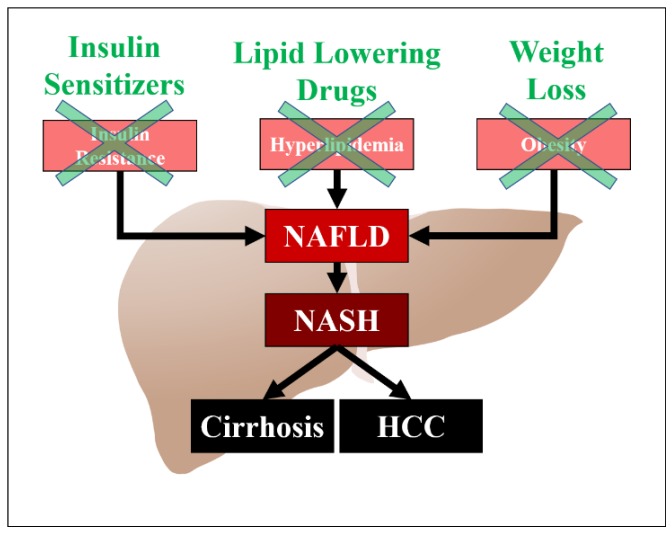
Pathogenesis of NAFLD/NASH and potential targets for treatment. HCC (Hepatocellular carcinoma).

Non-alcoholic fatty liver disease (NAFLD) is the most common cause of chronic liver disease in the developed world, and can progress to cirrhosis and hepatocellular carcinoma [1]. It is also the most common cause of elevated liver enzymes. Risk factors for NAFLD include obesity, diabetes mellitus, and hyperlipidemia. Obesity is also a significant risk factor for NAFLD, independent of hyperlipidemia, and weight loss has a beneficial effect on the prevention of hepatic fibrosis [2]. Patients with NAFLD are predisposed to cardiovascular mortality with studies demonstrating up to a twofold increase in the risk of cardiovascular disease in this population [3]. There are several hypotheses regarding the pathogenesis of NAFLD, which include insulin resistance, oxidative stress, and lipotoxicity (Figure 1). Hyperlipidemia is a significant risk factor for NAFLD and associated cardiovascular disease. The precise mechanism of action and the exact pathogenetic pathway on how hyperlipidemia increases the risk of NAFLD has not been elucidated, but may be related to an increased accumulation of lipids in the hepatocytes. Major sources of fatty acid delivery to hepatocytes include splanchnic lipolysis of visceral fat, lipogenesis, and the ingestion of fatty foods. Insulin resistance also downregulates low density lipoprotein (LDL)-receptor expression, leading to elevated levels of LDL. Multiple studies have shown the efficacy of lipid-lowering drugs in patients with NAFLD and its subset non-alcoholic steatohepatitis (NASH). The aim of this article is to review the medical literature on the utility of different anti-hyperlipidemic drugs in patients with NAFLD.

## 2. Lipid-Lowering Drugs

### 2.1. Statins

Statins are the most widely used lipid lowering drugs due to their known efficacy in reducing cardiovascular mortality in patients with coronary artery disease and diabetes mellitus. These beneficial effects are not only due to the cholesterol-lowering ability of statins, but also due to their anti-inflammatory, vasodilatory (statins are nitric oxide donor), vascular remodeling, and anti-fibrotic effects independent of cholesterol-lowering activity. As inflammatory mechanisms are involved in the pathogenesis of NAFLD/NASH, statins have shown promising results decreasing liver fibrosis in patients with NAFLD. Generally, statins have been shown to be safe and effective treatment options for the indication of dyslipidemia in the context of NAFLD with the exception of Child-Pugh B and C cirrhosis and in particular if the total bilirubin level is greater than 3 mg/dL [4,5]. Table 1 summarizes the current studies evaluating the utility of different statins in NAFLD and NASH.

### 2.2. Simvastatin

Multiple studies have shown safety and efficacy results of simvastatin in patients with NAFLD and NASH. Abel et al. conducted a study on a small group of NAFLD patients and revealed that simvastatin at 20 mg/day for 6 months significantly decreased aspartate aminotransferase (AST), alanine aminotransferase (ALT), and LDL cholesterol levels in these patients [6]. Liver histological evaluation was not done in this study. In another randomized controlled trial, simvastatin at 40 mg/day for 12 months was compared with a placebo, which significantly reduced LDL cholesterol levels [7]. Although patient cholesterol levels were lowered in the simvastatin group, no significant differences were found in levels of transaminases or hepatic fibrosis.

### 2.3. Atorvastatin

Several clinical studies reported the safety and efficacy of atorvastatin in NAFLD/NASH patients [8,9,10,11,12]. A randomized controlled trial comparing atorvastatin with the combination of atorvastatin and fenofibrate or fenofibrate alone revealed that atorvastatin alone or in combination with fenofibrate could improve liver enzymes and ultrasonographic features of NAFLD [8]. In a St. Francis Heart study, atorvastatin along with vitamins C and E was associated with reduced likelihood of developing hepatic steatosis in patients with NAFLD [9]. Although the results of this study were promising, they may be confounded by the use of vitamin E, as vitamin E alone has been shown to be beneficial in NAFLD patients [19]. In a study by Gomez-Dominguez, 22 hyperlipidemic NAFLD patients received 10–80 mg/day of atorvastatin for 6 months and showed significant improvement in aminotransferase and cholesterol levels [10]. Kimura et al. reported the role of advanced glycation end products (AGEs) in NASH, as the levels of these products were significantly high in NASH patients. Treatment of patients with atorvastatin resulted in a significant decrease in AGE levels and an improvement in metabolic parameters related to NASH [11]. In a study done on 27 biopsy-proven NASH patients with hyperlipidemia, Kiyci et al. revealed the promising effects of atorvastatin 10 mg/day for 6 months [12]. Atorvastatin was shown to decrease aminotransferase activity and significantly decrease fatty infiltration of the liver. These studies favor the beneficial effects of atorvastatin in NAFLD patients.

### 2.4. Pravastatin

The current evidence regarding the use of pravastatin in NAFLD patients is lacking. In a study on five patients with biopsy-proven steatohepatitis, Rallidis et al. revealed that the use of pravastatin resulted in an improvement of hepatic histological findings [13]. Further studies—especially randomized controlled trials—are needed to further investigate the utility of pravastatin in NAFLD patients.

### 2.5. Pitavastatin

Pitavastatin has been indicated to be beneficial in inhibiting hepatic fibrosis in NASH rat models, but research on its utility in patients with NAFLD is still too premature to make any conclusions. Hyogo et al. conducted an open-label pilot study that revealed 2 mg/day of pitavastatin for 12 months decreased the severity of hepatic steatosis and NASH-related parameters [14]. Although the study results are promising, further studies are necessary to support these findings.

### 2.6. Lovastatin

In a multicentric prospective study, 10 mg/day of lovastatin for four months was shown to significantly decrease transaminases, cholesterol levels, and aminotransferase to platelet ratio indices [15]. Again, more studies are needed to support the beneficial effects of lovastatin in NAFLD patients.

### 2.7. Rosuvastatin

In a preliminary report on six patients with NASH, 10 mg/day of rosuvastatin for 12 months not only showed improvement in AST and ALT levels, but also the complete resolution of ultrasonographic findings of NASH in five patients [16]. Similar beneficial effects were seen in a prospective study on 20 patients, in which 10 mg/day of rosuvastatin for 12 months resulted in the complete resolution of ultrasonographic findings of NASH in 19 patients [17]. Nakahara et al. also revealed beneficial effects of 2.5 mg/day of rosuvastatin for 24 months, and showed an improvement of NASH-related parameters and histological features in some patients [18].

### 2.8. Non-Statin Lipid Lowering Drugs

Table 2 summarizes the current evidence regarding the utility of lipid-lowering drugs other than statins in patients with NAFLD and NASH. 

### 2.9. Fibrates

Fibrates or fibric acid derivatives are used to treat hypertriglyceridemia and primary hypercholesterolemia, mainly by activating peroxisome proliferator-activated receptor alpha (PPAR-alpha). Multiple studies have been done to evaluate the safety and efficacy of these agents in treating hyperlipidemia in NAFLD/NASH patients [8,20,21,22]. In a prospective open-label randomized study, Athyros et al. included 186 patients that were randomly assigned to receive either 20 mg/day of atorvastatin, 200 mg/day of fenofibrate, or both [8]. A complete resolution of biochemical and ultrasonographic evidence of NAFLD was seen in 42% of the patients in the fenofibrate group compared to 67% of patients taking atorvastatin alone, and 70% in the combination therapy group [8]. In a randomized controlled trial (RCT), Basarangolu et al. included 23 patients taking 600 mg/day of gemfibrozil for four weeks, and compared these with 23 placebo patients who were not treated with any lipid-lowering drugs. Patients in the gemfibrozil group had significantly lower levels of AST, ALT, and GGT without any significant changes in liver histology [20]. Laurin et al. evaluated the utility of the fibric acid derivative clofibrate in 16 NASH patients with hyperlipidemia taking 2 g /day [21]. The study did not find any improvement in aminotransferases, GGT, or bilirubin levels. In a pilot study on 16 biopsy-proven NAFLD patients, Fernandez-Miranda et al. revealed that 200 mg/day of fenofibrate for 48 weeks resulted in decreased insulin resistance, aminotransferases levels, and signs of metabolic syndrome [22]. Although the drug showed promising results in decreasing the proportion of patients with metabolic syndrome, its effect on liver histology was minimal. Hence, considering current evidence, we conclude that the utility of fibrates and fibric acid derivatives in ameliorating the histological features of NAFLD is still unclear and requires further studies. 

### 2.10. Elafibranor

Elafibranor is an activator of both Peroxisome proliferator-activated receptor (PPAR)-α and PPAR-δ that has anti-inflammatory effects and helps to improve lipid metabolism and insulin resistance. In a randomized controlled double-blind trial, 276 patients with NASH were divided into three groups to receive either 80 mg/day of elafibranor, 120 mg/day of elafibranor, or a placebo for 52 weeks [23]. Aminotransferase activity, glucose levels, and inflammatory markers were significantly lower in the elafibranor 120 mg group compared to the placebo group. A post-hoc analysis of the study also revealed that elafibranor (120 mg/d for 1 year) resolved NASH without fibrosis worsening in the greater proportion of patients compared to placebo, but there was no difference in the outcome in the intention-to-treat analysis [23]. These results are encouraging for the use of elafibranor in NASH patients, but require further studies—especially randomized controlled trials—to further strengthen the evidence of these beneficial effects.

### 2.11. Niacin

Niacin (nicotinic acid or vitamin B-3) is used to treat vitamin deficiency (pellagra) and has lipid-lowering effects. The mechanism by which these drugs exert their lipid-lowering effects is still unclear. Fabbrini et al. reported that the combination of 200 mg/day of fenofibrate for 8 weeks and extended-release niacin at 2000 mg/day for 16 weeks lowered the plasma levels of VLDL-triglycerides, but did not alter intrahepatic triglyceride content [24]. The safety and efficacy of niacin in NAFLD patients has yet to be established, and requires further clinical studies.

### 2.12. Ezetimibe

Ezetimibe exerts its lipid-lowering effects by inhibiting small intestinal absorption of cholesterol via its effects on the sterol transporter Niemann–Pick C1-Like1 (NPC1L1). These molecules are also expressed in the liver, and play a role in hepatic cholesterol accumulation. The combination of ezetimibe and statins has been shown to improve LDL cholesterol levels in patients with hypercholesterolemia. In one study, Yoneda et al. reported 10 mg/day of ezetimibe for 6 months in NASH patients with hyperlipidemia resulted in significant improvement in histological findings, NAFLD activity scores, and steatosis in the liver [25]. They also revealed improvement in AST, ALT, GGT, LDL cholesterol levels, and C-reactive protein (CRP) in these patients. Similar results were seen in a study done by Park et al. regarding 45 patients with biopsy-proven NAFLD [26]. They reported that 10 mg/day of ezetimibe for 24 months improved the biochemical and histological abnormalities of NAFLD. Chan et al. also reported that weight loss along with ezetimibe was associated with an improvement in hepatic steatosis [27]. These study results favor the use of ezetimibe in NAFLD/NASH patients with hyperlipidemia, as it might ameliorate the biochemical and histological features of NAFLD.

### 2.13. Omega-3 (n-3) Polyunsaturated Fatty Acids (PUFAs)

The use of n-3 PUFAs increases the levels of adiponectin in the blood and decreases serum levels of triglycerides, leptin, and insulin, which promote weight loss and improve insulin resistance [28,29]. In a study of 40 patients with NAFLD, Spadro et al. reported improvement in serum AST, ALT, triglyceride levels, and fatty liver with the use of n-3 PUFAs [28]. Similar beneficial effects were seen in a study by Capanni et al., which reported that n-3 PUFA supplementation of 1 g/day for 12 months resulted in the improvement of AST, ALT, GGT, triglycerides, and ultrasonographic features of hepatic steatosis [29]. Current evidence is therefore encouraging, but further studies are needed to define the utility of n-3 PUFAs for management of NAFLD.

## 3. Conclusions

Statins have been shown to be most beneficial in the prevention of hepatic fibrosis in patients with NAFLD/NASH. Hence, in light of current evidence, we recommend considering statins and vitamin E along with weight loss and exercise in these patients. Although evidence regarding the utility of lipid-lowering drugs in patients with NAFLD/NASH is convincing, with most studies reporting that lipid-lowering drug use showed an improvement in hepatic steatosis, formal guidelines are lacking in this regard—mainly due to the absence of larger randomized controlled trials. Despite the need for more randomized controlled trials regarding these drugs, our review will help guide clinicians in prescribing lipid-lowering agents in NAFLD patients. This, in turn, may help decrease the morbidity and mortality associated with this serious disease. 

## Figures and Tables

**Table 1:** Studies evaluating the utility of Statins in non-alcoholic fatty liver disease (NAFLD)/ non-alcoholic steatohepatitis (NASH).

Author’s Name and Year of Publication	Drug(s) Used	Dose	Patient Population and Sample Size	Duration of Therapy	Outcome
Abel et al. [6] 2009	Simvastatin	20 mg/day	45 patients with NAFLD secondary to diabetes and metabolic syndrome	6 months	Decrease in aspartate aminotransferase (AST), and alanine aminotransferase (ALT) and low density lipoprotein (LDL) cholesterol. Liver histology was not evaluated.
Nelson et al. [7] 2009	Simvastatin	40 mg/day	16 patients with biopsy-proven NASH	12 months	No significant difference from the improvement of transaminases or hepatic fibrosis. A 26% reduction in LDL levels was seen in the simvastatin group.
Athyros et al. [8] 2006	Atorvastatin and/or Fenofibrate	20 mg/day alone or in combination with fenofibrate 200 mg/day	186 non-diabetic patients with NAFLD and metabolic syndrome	54 weeks	67% of patients on atorvastatin, 42% on fenofibrate, and 70% on combination treatment no longer had biochemical and ultrasonographic evidence of NAFLD.
Foster et al. [9] 2011	Atorvastatin	20 mg/day along with vitamin C 1g, and vitamin E 1000 IU	1005 healthy men and women between ages 50–70	Mean follow-up 3.6 years	Reduced odds of having hepatic steatosis in NAFLD patients by 71%.
Gómez-Domínguez et al. [10] 2006	Atorvastatin	10–80 mg/day	25 patients with NAFLD (only 22 completed the study)	6 months	Significant improvement in aminotransferase activities and cholesterol levels.
Kimura et al. [11] 2010	Atorvastatin	10 mg/day	43 NASH patients with dyslipidemia	12 months	Improvement in metabolic parameters related to NASH.
Kiyci et al. [12] 2003	Atorvastatin	10 mg/day	44 patients with NASH	6 months	Decrease in aminotransferase activity and fatty infiltration of the liver.
Rallidis et al. [13] 2004	Pravastatin	20 mg/day	5 patients with steatohepatitis and liver enzyme abnormalities	6 months	Improvement in histologic findings of steatohepatitis.
Hyogo et al. [14] 2011	Pitavastatin	2 mg/day	20 NASH patients with dyslipidemia	12 months	Decrease in severity of hepatic steatosis. Fibrosis stage improved in 42% of the patients.
Mihaila et al. [15] 2009	Lovastatin	10 mg/day	87 patients with NASH and dyslipidemia	4 months	Decrease in aminotransferase and cholesterol levels.
Kargiotis et al. [16] 2014	Rosuvastatin	10 mg/day	6 non-diabetic patients with NASH, metabolic syndrome, and dyslipidemia	12 months	Resolution of ultrasonographic findings of NASH. Improvement in aminotransferases; ALT and AST levels were reduced by 76% and 61%, respectively.
Kargiotis et al. [17] 2015	Rosuvastatin	10 mg/day	20 patients with NASH, metabolic syndrome, and dyslipidemia	12 months	Resolution of ultrasonographic findings of NASH.
Nakahara et al. [18] 2012	Rosuvastatin	2.5 mg/day	19 patients with NASH and dyslipidemia	24 months	Improvement of NASH-related parameters, with 33.3% patients showing improvement in non-alcoholic fatty liver disease activity score and fibrotic stage.

**Table 2:** Studies evaluating the utility of lipid-lowering drugs in NAFLD/NASH.

Author’s Name and Year of Publication	Drug(s) Used	Dose	Patient Population and Sample Size	Duration of Therapy	Outcome
Athyros et al. [8] 2006	Fenofibrate alone or in combination with atorvastatin	200 mg/day alone or in combination with atorvastatin 20 mg/day	186 non-diabetic patients with NAFLD and metabolic syndrome	54 weeks	42% of the patients in the fenofibrate-only treatment group had a complete resolution of biochemical and ultrasonographic evidence of NAFLD.
Basarangolu et al. [20] 1999	Gemfibrozil	600 mg/day	46 patients with NASH and liver enzyme abnormalities	4 weeks	Lower levels of ALT, AST, and gamma glutamyl transferase (GGT). No change in liver histology.
Laurin et al. [21] 1996	Clofibrate	2 g/day	40 patients with NASH	12 months	No improvement in aminotransferase activities.
Fernandez-Miranda et al. [22] 2008	Fenofibrate	200 mg/day	16 NAFLD patients	48 weeks	Decrease in insulin resistance, aminotransferases levels, and signs of metabolic syndrome. No significant effect on liver histology.
Ratziu et al. [23] 2016	Elafibranor	120 mg/day	276 patients with NASH without cirrhosis	52 weeks	Resolution of NASH without fibrosis worsening in the greater proportion of patients compared to placebo (19% vs. 12%; odds ratio = 2.31; *p* = 0.045).
Fabbrini et al. [24] 2010	Niacin	2000 mg/day	27 obese patients with NAFLD	16 weeks	Lowered plasma levels of very-low density lipoprotein (VLDL)-TGs but no change in intrahepatic triglycerides (TGs) content.
Yoneda et al. [25] 2010	Ezetimibe	10 mg/day	10 patients with NASH and dyslipidemia	6 months	Improvement in hepatic steatosis and histological findings. 6 out of 10 patients showed improvement in their fibrosis stage.
Park et al. [26] 2011	Ezetimibe	10 mg/day	45 NAFLD patients	24 months	Improvement in biochemical and histological abnormalities of NAFLD.
Chan et al. [27] 2010	Ezetimibe	10 mg/day	25 patients with central obesity	16 weeks	Improvement in hepatic steatosis.
Spadro et al. [28] 2008	Omega-3 (n-3) polyunsaturated fatty acids (PUFAs)	2 g/day	40 patients with NAFLD	6 months	Improvement in serum AST, ALT, triglyceride levels, and fatty liver.
Capanni et al. [29] 2006	n-3 PUFAs	1 g/day	56 patients with NAFLD	12 months	Improvement of AST, ALT, GGT, triglycerides, and ultrasonographic features of hepatic steatosis.

## References

[1] Loomba R., Sanyal A.J. (2013). The global NAFLD epidemic. Nat. Rev. Gastroenterol. Hepatol..

[2] Li L., Liu D.-W., Yan H.-Y., Wang Z.-Y., Zhao S.-H., Wang B. (2016). Obesity is an independent risk factor for non-alcoholic fatty liver disease: Evidence from a meta-analysis of 21 cohort studies. Obes. Rev..

[3] Ballestri S., Lonardo A., Bonapace S., Byrne C.D., Loria P., Targher G. (2014). Risk of cardiovascular, cardiac and arrhythmic complications in patients with non-alcoholic fatty liver disease. World J. Gastroenterol..

[4] Sigler M.A., Congdon L., Edwards K.L. (2018). An evidence-based review of statin use in patients with nonalcoholic fatty liver disease. Clin. Med. Insights Gastroenterol..

[5] Magan-Fernandez A., Rizzo M., Montalto G., Marchesini G. (2018). Statins in liver disease: Not only prevention of cardiovascular events. Expert Rev. Gastroenterol. Hepatol..

[6] Abel T., Fehér J., Dinya E., Eldin M.G., Kovács A. (2009). Safety and efficacy of combined ezetimibe/simvastatin treatment and simvastatin monotherapy in patients with non-alcoholic fatty liver disease. Med. Sci. Monitor.

[7] Nelson A., Torres D.M., Morgan A.E., Fincke C., Harrison S.A. (2009). A pilot study using simvastatin in the treatment of nonalcoholic steatohepatitis: A randomized placebo-controlled trial. J. Clin. Gastroenterol..

[8] Athyros V.G., Mikhailidis D.P., Didangelos T.P., Giouleme O.I., Liberopoulos E.N., Karagiannis A., Kakafika A.I., Tziomalos K., Burroughs A.K., Elisaf M.S. (2006). Effect of multifactorial treatment on non-alcoholic fatty liver disease in metabolic syndrome: A randomised study. Curr. Med. Res. Opin..

[9] Foster T., Budoff M.J., Saab S., Ahmadi N., Gordon C., Guerci A.D. (2011). Atorvastatin and antioxidants for the treatment of nonalcoholic fatty liver disease: The St Francis heart study randomized clinical trial. Am. J. Gastroenterol..

[10] Gómez-Domínguez E., Gisbert J.P., Moreno-Monteagudo J.A., García-Buey L., Moreno-Otero R. (2006). A pilot study of atorvastatin treatment in dyslipemid, non-alcoholic fatty liver patients. Aliment. Pharm. Therap..

[11] Kimura Y., Hyogo H., Yamagishi S.-I., Takeuchi M., Ishitobi T., Nabeshima Y., Arihiro K., Chayama K. (2010). Atorvastatin decreases serum levels of advanced glycation endproducts (AGEs) in nonalcoholic steatohepatitis (NASH) patients with dyslipidemia: Clinical usefulness of AGEs as a biomarker for the attenuation of NASH. J. Gastroenterol..

[12] Kiyici M., Gulten M., Gurel S., Nak S.G., Dolar E., Savci G., Adim S.B., Yerci O., Memik F. (2003). Ursodeoxycholic acid and atorvastatin in the treatment of nonalcoholic steatohepatitis. Can. J. Gastroenterol..

[13] Rallidis L.S., Drakoulis C.K., Parasi A.S. (2004). Pravastatin in patients with nonalcoholic steatohepatitis: Results of a pilot study. Atherosclerosis.

[14] Hyogo H., Ikegami T., Tokushige K., Hashimoto E., Inui K., Matsuzaki Y., Tokumo H., Hino F., Tazuma S. (2011). Efficacy of pitavastatin for the treatment of non-alcoholic steatohepatitis with dyslipidemia: An open-label, pilot study. Hepatol. Res..

[15] Mihaila R.-G., Nedelcu L., Fratila O., Rezi E.-C., Domnariu C., Deac M. (2009). Effects of lovastatin and pentoxyphyllin in nonalcoholic steatohepatitis. Hepato-Gastroenterol..

[16] Kargiotis K., Niki K., Athyros V.G., Giouleme O., Patsiaoura K., Katsiki E., Mikhailidis D.P., Karagiannis A. (2014). Effect of rosuvastatin on non-alcoholic steatohepatitis in patients with metabolic syndrome and hypercholesterolaemia: A preliminary report. Curr. Vasc. Pharm..

[17] Kargiotis K., Athyros V.G., Giouleme O., Katsiki N., Katsiki E., Anagnostis P., Boutari C., Doumas M., Karagiannis A., Mikhailidis D.P. (2015). Resolution of non-alcoholic steatohepatitis by rosuvastatin monotherapy in patients with metabolic syndrome. World J. Gastroenterol..

[18] Nakahara T., Hyogo H., Kimura Y., Ishitobi T., Arihiro K., Aikata H., Takahashi S., Chayama K. (2012). Efficacy of rosuvastatin for the treatment of non-alcoholic steatohepatitis with dyslipidemia: An open-label, pilot study. Hepatol. Res..

[19] Sanyal A.J., Chalasani N., Kowdley K.V., McCullough A., Diehl A.M., Bass N.M., Neuschwander-Tetri B.A. (2010). Pioglitazone, vitamin E, or placebo for nonalcoholic steatohepatitis. New Engl. J. Med..

[20] Basaranoglu M., Acbay O., Sonsuz A. (1999). A controlled trial of gemfibrozil in the treatment of patients with nonalcoholic steatohepatitis. J. Hepatol..

[21] Laurin J., Lindor K.D., Crippin J.S., Gossard A., Gores G.J., Ludwig J., Rakela J., McGill D.B. (1996). Ursodeoxycholic acid or clofibrate in the treatment of non-alcohol-induced steatohepatitis: A pilot study. Hepatology.

[22] Fernández-Miranda C., Pérez-Carreras M., Colina F., López-Alonso G., Vargas C., Solís-Herruzo J.A. (2008). A pilot trial of fenofibrate for the treatment of non-alcoholic fatty liver disease. Dig. Liver Dis..

[23] Ratziu V., Harrison S.A., Francque S., Bedossa P., Lehert P., Serfaty L., Romero-Gomez M., Boursier J., Abdelmalek M., Caldwell S. (2016). Elafibranor, an agonist of the peroxisome proliferator-activated receptor-α and -δ, induces resolution of nonalcoholic steatohepatitis without fibrosis worsening. Gastroenterology.

[24] Fabbrini E., Mohammed B.S., Korenblat K.M., Magkos F., McCrea J., Patterson B.W., Klein S. (2010). Effect of fenofibrate and niacin on intrahepatic triglyceride content, very low-density lipoprotein kinetics, and insulin action in obese subjects with nonalcoholic fatty liver disease. J. Clin. Endocrinol. Metab..

[25] Yoneda M., Fujita K., Nozaki Y., Endo H., Takahashi H., Hosono K., Suzuki K. (2010). Efficacy of ezetimibe for the treatment of non-alcoholic steatohepatitis: An open-label, pilot study. Hepatol. Res..

[26] Park H., Shima T., Yamaguchi K., Mitsuyoshi H., Minami M., Yasui K., Itoh Y., Yoshikawa T., Fukui M. (2011). Efficacy of long-term ezetimibe therapy in patients with nonalcoholic fatty liver disease. J. Gastroenterol..

[27] Chan D.C., Watts G.F., Gan S.K., Ooi E.M.M., Barrett P.H.R. (2010). Effect of ezetimibe on hepatic fat, inflammatory markers, and apolipoprotein B-100 kinetics in insulin-resistant obese subjects on a weight loss diet. Diabetes Care.

[28] Spadaro L., Magliocco O., Spampinato D., Piro S., Oliveri C., Alagona C., Papa G., Rabuazzo A.M., Purrello F. (2008). Effects of *N*-3 polyunsaturated fatty acids in subjects with nonalcoholic fatty liver disease. Dig. Liver Dis..

[29] Capanni M., Calella F., Biagini M.R., Genise S., Raimondi L., Bedogni G., Svegliati-Baroni G., Sofi F., Milani S., Abbate R. (2006). Prolonged *N*-3 polyunsaturated fatty acid supplementation ameliorates hepatic steatosis in patients with non-alcoholic fatty liver disease: A pilot study. Aliment. Pharm. Therap..

